# Rapid diagnosis of tuberculosis using Xpert MTB/RIF assay - Report from a developing country 

**DOI:** 10.12669/pjms.311.6970

**Published:** 2015

**Authors:** Shagufta Iram, Asyia Zeenat, Shahida Hussain, Noshin Wasim Yusuf, Maleeha Aslam

**Affiliations:** 1 Shagufta Iram, M.Phil Microbiology, Assistant Professor of Pathology, Allam Iqbal Medical College, Lahore, Pakistan.; 2 Asyia Zeenat, MSc Molecular Biology, Allam Iqbal Medical College, Lahore, Pakistan.; 3 Shahida Hussain, M.Phil Biotechnology, Allam Iqbal Medical College, Lahore, Pakistan.; 4 Noshin Wasim Yusuf, MBBS, M.Phil, MRC.Path, FRC.Path, Professor and Head of Department of Pathology, Allam Iqbal Medical College, Lahore, Pakistan.; 5 Maleeha Aslam, M.Phil Microbiology, Allam Iqbal Medical College, Lahore, Pakistan.

**Keywords:** Tuberculosis, Gene-Xpert, TB culture, ZN smear

## Abstract

**Objective:**

To evaluate the diagnostic accuracy of the Xpert MTB/RIF assay for the detection of *M. tuberculosis* in pulmonary and extrapulmonary specimens and to compare it with conventional techniques.

**Methods:**

During a period of 10 months from December 2012 through September 2013, two hundred and forty five clinically TB suspects were enrolled for Xpert MTB\RIF assay. The cohort comprised of 205 suspects of pulmonary TB and 40 of extrapulmonary TB (EPTB). The 40 EPTB samples included pus aspirated from different sites of the body (n=19), pleural fluid (n=11), ascitic fluid (n=7), pericardial fluid, CSF and urine one each. Ziehl-Neelsen (ZN) Stained smear microscopy, culture on LJ media and Xpert MTB/RIF assay was performed on samples from these patients.

**Results:**

*M. tuberculosis* (*MTB*) were detected by Xpert MTB/RIF test in 111 (45.3%) out of 245 samples. Of these, 85 (34.7%) were smear positive on ZN staining and 102 (41.6%) were positive on LJ cultures. Rifampicin resistance was detected in 16 (6.5%) patients. Nine out of 19 pus samples (47.3%) were positive for MTB by Gene Xpert, 03 (15.8%) on ZN staining and 04 (21%) on LJ culture. MTB could not be detected in any other extrapulmonary sample.

**Conclusion:**

Xpert MTB/RIF is a sensitive method for rapid diagnosis of Tuberculosis, especially in smear negative cases and in EPTB as compared to the conventional ZN staining. Among EPTB cases the highest yield of positivity was shown in Pus samples. For countries endemic for TB GeneXpert can serve as a sensitive and time saving diagnostic modality for pulmonary and EPTB.

## INTRODUCTION

An alarming rise in the global incidence of infections caused by *Mycobacterium tuberculosis(MTB) *has prompted the need for rapid diagnostic techniques.^1^ With an estimated 8.7 million new cases and 1.4 million deaths every year, tuberculosis (TB) remains a leading public health problem worldwide.^2 ^Over 90% of TB cases develop among people living in low and middle income countries like Pakistan. Based on worldwide incident cases in 2007, World Health Organization (WHO) ranked Pakistan eighth in the list of high burden countries.^3^ EPTB is equally prevalent in all the high burden zones. According to WHO, 34,000 (15%) of newly reported TB cases in 2007 were extrapulmonary.^4^^,^^5^

Inability to rapidly diagnose and treat the affected patients leads to increased morbidity and mortality, development of secondary resistance (including extensively drug-resistant tuberculosis) and ongoing transmission of the disease.^6^ In this situation, not only rapid TB case detection, but also the early determination of MDR status is important. Conventionally the diagnosis of pulmonary tuberculosis has been based on clinical scenario, chest X-ray findings, smear microscopy for acid fast bacillus, or bacterial isolation by culture. In developing countries, out of all the lab investigations, diagnosis still relies heavily on the use of smear microscopy, which has a low sensitivity and specificity as compared to the culture. The microbiological identification of *M.tuberculosis *by culture remains the gold standard for diagnosis of tuberculosis. However, the conventional culture technique for Mycobacteria does not provide a rapid diagnosis, is a cumbersome procedure and requires sophisticated laboratory facilities of biological safety lab level II/III that cannot be afforded in most of resource limited settings.^7^^,^^8^ As an alternate, recent molecular diagnostic techniques are increasingly being promoted owing to their rapid turnaround time and high sensitivity and specificity.^9^

The World Health Organization (WHO) has endorsed the implementation of GeneXpert MTB/RIF assay for national tuberculosis programs in developing countries.^10^ The Xpert MTB/RIF (Cepheid Inc.) is an automated, user friendly and rapid test based on nested real-time PCR assay and molecular beacon technology for MTB detection and RIF resistance. The results are obtained within a short period of 2 hours.^1^ Further on, the technique is not prone to cross-contamination, requires minimal Biosafety facilities and has a high sensitivity in smear-negative pulmonary TB.^7^^,^^11^^-^^13^ Its effectiveness in EPTB is also documented.^7^^,^^9^^,^^14^ The diagnosis of EPTB is often difficult to establish, considering that number of bacteria in specimens is often very low, a collection often requires invasive procedures, and it is not easy to obtain multiple samples. In this scenario GeneXpert is a potentially useful tool for extrapulmonary specimens.^14^

The purpose of this study was to evaluate the sensitivity and specificity of the Xpert MTB/RIF assay for the detection of *M. tuberculosis* in pulmonary as well as extrapulmonary specimens and to compare it with conventional techniques for TB diagnosis.

## METHODS

A total of 245 patients were included in this study during a period of 10 months from December 2011 through September 2013. The cohort was comprised of pulmonary (n=205) and extrapulmonary (n=40) TB suspects. Inclusion of 205 pulmonary TB suspects was based on clinical symptoms (productive cough for more than two weeks, persistent low-grade fever, night sweat and weight loss) and radiological findings consistent with tuberculosis. Sputum samples were collected from all these cases. The 40 EPTB suspects were selected on the basis of clinical presentation, radiological findings and histo-pathological evidence. The extrapulmonary samples (n=40) were comprised of aspirated us from different sites (n=19), pleural fluid (n=11), ascitic fluid (n=07), pericardial fluid, urine and CSF one each.

***Processing of samples:***

***A. Sputum: ***All the sputum samples were subjected to

1. ZN staining for smear microscopy following the WHO recommended protocol.^15^

2. Xpert MTB/RIF assay: Sputum samples were processed directly from Xpert MTB/RIF test, according to manufacturer’s protocol. Sample reagent was added in a 2:1 ratio to unprocessed sputum in 15 ml falcon tube and the tube was manually agitated twice during a 15 minute incubation period at room temperature. Then 2 ml of the inactivated material was transferred to the test cartridge by a sterile disposable pipette (provided with kits). Cartridges were loaded into the GeneXpert. The interpretation of data from MTB/RIF tests was software based and not user dependent.^8^

3. Culture on Lowenstein–Jensen (L.J) media: Culture was put up after decontamination of the sputum samples on LJ media slopes following the standard protocol.^16^ LJ culture was used as the reference method in our study.

***B. Extrapulmonary samples: ***EPTB samples were concentrated by cytocentrifugation at 3000g for 20 minutes and the deposit was processed as for sputum sample using, ZN staining, Xpert MTB/RIF assay and culture on L.J media.

The sensitivity and specificity of each test were calculated according to following formula.

*Sensitivity:*

$\frac{\mathrm{True positive}(\mathrm{TP})}{\mathrm{True positive}(\mathrm{TP})+\mathrm{False negative}(\mathrm{FN})}\times 100$

*Specificity:*

$\frac{\mathrm{True negative}(\mathrm{TN})}{\mathrm{True negative}(\mathrm{TN})+\mathrm{False positive}(\mathrm{FP})}\times 100$

**Table-I:** Overall results of Gene Xpert

*Results *	*Pulmonary *	*Extrapulmonary*	*Total*
No. of samples	205	40	245
MTB Detected	102 (49.8%)	9 (22.5%)	111
Rifampicin resistance Detected	16 (15.7%)	0	16

## RESULTS

Out of the total 245 samples (205 pulmonary TB, 40 EPTB) *Mycobacterium tuberculosis (MTB)* was detected by Xpert MTB/RIF assay in 111 (45.3%), 102 (49.8%) being pulmonary TB suspects and 9 (22.5%) EPTB suspects (Table-I). Comparison of results of pulmonary samples amongst the 3 techniques utilized in this study is depicted in Table-II. It was observed that out of 205 pulmonary samples, 82 (40%) were positive on ZN microscopy, 98 (47.8%) on culture and 102 (49.7%) with Gene Xpert (Table-II). A similar trend was observed with EPTB samples where Xpert detected MTB in 09 (22.5%) out of 40 cases, whereas only 04 (10%) and 03 (7.5%) cases were positive on LJ culture and ZN smear respectively. It was observed that GeneXpert could detect 12.5% and 15% additional positive cases as compared to LJ culture and ZN microscopy respectively in EPTB (Table-II). There is a significance difference found between ZN and LJ culture for EPTB (P<0.05).

Comparison of the results of ZN smear microscopy with GeneXpert revealed that MTB was detected in all the ZN smear positive sputum samples (n=82) by GeneXpert technique. Similarly GeneXpert could detect MTB in all the samples (n=98) which were positive for MTB on L.J culture (Table-III). The GeneXpert was able to pick up 16 additional cases which were negative on ZN smear microscopy, depicting its utility in diagnosing smear negative TB cases (Table-III).

All cases with positive radiological, histological and bacteriological evidence for TB were referred for anti-tuberculosis therapy to the treating physician. The cohort was followed up after two months for assessing the response of treatment. 

Pulmonary samples were subdivided into two categories: Pulmonary TB suspects (n=75) and MDR (Multi Drug Resistance) suspects (n=130) (Table-IV). The latter comprised of all treatment categories i.e. Relapse cases (n=89), defaulters (n=25) and treatment failures (n=16). It was observed that in both the groups the sensitivity of detecting MTB using Xpert exceeded by 10% as compared to ZN smear microscopy and that rifampicine resistance was detected in none of pulmonary TB suspects. However, in MDR suspects rifampicine resistance was detected in 16 out of 74 (21.6%) MTB positive cases (Table-IV) which was confirmed by drug susceptibility testing (DST).

## DISCUSSION

Conventional laboratory techniques as ZN smear microscopy for diagnosis of tuberculosis from clinical specimens is less sensitive as compared to the culture because large bacillary load (10^5^/ml) will be required for a smear to become positive.^17^

Moreover the conventional cultures are time consuming and require Biosafety setup and trained laboratory personnels.^9^ The GeneXpert MTB/RIF assay is a rapid molecular biology/gene based assay that can be used close to the point of care by operators with minimal technical expertise. The technique enables diagnosis of TB and simultaneous assessment of rifampicin resistance to be completed within 2 hour.^1^ The extra advantage is the convenience of sample processing where unprocessed sputum samples as well as clinical specimens from extrapulmonary sites can be directly assayed.^17^

In the present study, we have evaluated the diagnostic accuracy of Xpert MTB/RIF assay both for pulmonary and EPTB cases and compared it with the conventional techniques. Out of 245 TB suspects 111 (45.3%) were Xpert positive which included 85 (76.6%) ZN smear positive and 26 (23.4%) smear negative cases. Here, the on time Xpert MTB/RIF assay could diagnose an additional 23.4% case along with an added advantage of much lower turnaround time. Bates, et* al. *in their study reported a better detection rate with Xpert when compared to smear microscopy and culture.^18^ However, compared to our study where 100% of the smear and culture positive cases (Table-V) were positive by Gene Xpert, they observed positive results in 95% cases only on Gene Xpert.^18^ In concordance with this study Zeka *et al*. also reported 100% specificity of MTB/RIF test in 110 clinically and microbiologically diagnosed tuberculosis patients.^13^

For pulmonary samples with all 3 techniques utilized in this study, we observed that out of 205 samples, 82 (40%) were positive on ZN microscopy, 98 (47.8%) on culture and 102 (49.7%) with GeneXpert. This indicates that Gene Xpert is a highly sensitive technique for MTB detection compared to the conventional techniques. We observed 100% sensitivity and specificity in smear positive and culture positive sputa with Xpert assay (Table-V). A further benefit was obtained in culture negative cases where an additional 4 cases were picked up by Gene Xpert. These results are in agreement with those reported in several previous studies^7^^,^^9^^,^^14^^,^^19^

We also evaluated the sensitivity and specificity of Gene Xpert in smear negative and smear positive cases irrespective of their culture status. We found 100% sensitivity and specificity for smear positive cases, whereas for smear negative the sensitivity and specificity was 80% and 96% respectively (Table-V). Zeka and coauthors in a similarly conducted study reported a much lower sensitivity (68.6%) in smear negative sputum samples with the specificity being 100%.While for smear positive cases their results matched ours (100% sensitive and specific).^13^ A low sensitivity range (72-75%) for smear negative cases has been reported in several previous studies as well.^1^^,^^8^^,^^11^^,^^20^^,^^21^ Comparing with our results Boehme *et al.* in their study reported 77% sensitivity of Xpert MTB/RIF assay in smear negative samples.^8^ In three other studies conducted in Spain, France and Netherlands a comparable performance was noted by testing pulmonary samples.^11^^,^^21^^,^^22^ Sensitivities for smear-positive TB were 98-100%^21^^,^^23^ and ranged between 57 and 83% for smear negative pulmonary TB.^11^^,^^13^^,^^20^^-^^22^^,^^24^ In the present study we observed 100% sensitivity and 96% specificity for sputum samples which is comparable with the above mentioned studies.

Comparable efficacy was observed for EPTB samples in our study. Here Xpert could detect 12.5% and 15% more positive cases as compared to L.J culture and ZN microscopy respectively indicating a higher sensitivity. In agreement, Hillemann *et al*. In their study of EPTB samples reported a sensitivity and specificity of 77.3% and 98.2%, respectively again indicating a higher sensitivity of Xpert as it could detect some of the culture negative cases as well.^9^ We observed a sensitivity of 100% and specificity of 86%, which was comparatively higher than the sensitivity observed by Zeka *et al*. which was 52% of EPTB samples.^13^ In another published work carried out in EPTB cases, Tortoli et al. reported 86.9% sensitivity and 99.7% specificity by GeneXpert which was equivalent to our results.^24^ The low sensitivity of culture (10%) as compared to that of Xpert (22%) may be attributed to; Paucibacillary nature of extrapulmonary specimens with uneven distribution of the bacilli and formation of clumps. Moreover, during NALC-NaOH decontamination process, there are more chances of killing of viable bacteria as compared to sample processing for Xpert assay in which better homogenization and liquefaction of samples is achieved.^25^

The Xpert MTB/RIF assay is a useful addition to the diagnostic armamentarium for rapid diagnosis of both pulmonary TB and EPTB as it has greatly shortened the time of detection up to two hours as compared to other techniques.^14^^,^^23^ This advantage is translated into clinical management for patients with smear negative TB as the Xpert assay reduces the time to start treatment for several weeks to just a few days.^17^^,^^26^

**Table-II:** Comparative results of ZN smear, LJ media and Gene-Xpert.

*Sample type*	*ZN smear +ve*	*Culture +ve*	*Xpert +ve*
Pulmonary samples (n=205)	82 (40%)	98 (47.8%)	102 (49.7%)
Extrapulmonary samples (n=40)	3 (7.5%)	4 (10%)	9 (22.5%)
Total (245)	85	102	111

**Table-III:** Comparison of ZN Smear with LJ culture and Gene Xpert method

*Smear results*	*Results of LJ culture and Gene-Xpert*			
	*L.J culture &Xpert +ve*	*L.J culture –ve* *Xpert +ve*	*L.J culture –ve* *Xpert –ve*	*Total*
ZN smear positive	82	0	0	82
ZN smear negative	16	4	103	123
Total	98	4	103	205

**Table-IV:** Results of two groups of pulmonary samples

*Test *	*PTB suspects (n=75)*	*MDR suspects (n=130)*
ZN smear +ve	20 (26.6%)	62 (47.7%)
L.J culture +ve	28 (37.3%)	70 (53.8%)
Xpert +ve	28 (37.3%)	74 (57%)
Rifampicin resistance +ve	None	16 (21.6%)

**Table-V:** Gene Xpert results compared to smear and LJ culture in sputum samples

	*Smear-Positive*		*Smear-Negative*		*Total*
	*Culture + ive*	*Culture – ive*	*Culture + ive*	*Culture – ive*	
Xpert-Positive	82	0	16	4	102
Xpert-Negative	0	0	0	103	103
Total	82	0	16	107	205
Sensitivity	100%		80%		
Specificity	100%		96%		

**Table-VI:** GeneXpert results compared to LJ culture in sputum samples

		*LJ Culture*		*Total*	*Sensitivity*	*Specificity*
		*Positive*	*Negative*			
GeneXpert	Positive	98	4	102	100%	96%
	Negative	0	103	103		
	Total	98	107	205		

**Table-VII:** *GeneXpert results compared to LJ culture in EPTB samples*

		*LJ Culture*		*Total*	*Sensitivity*	*Specificity*
		*Positive*	*Negative*			
GeneXpert	Positive	4	5	9	100%	86%
	Negative	0	31	31		
	Total	4	36	40		

## CONCLUSION

The Xpert MTB test is sensitive and specific for rapid diagnosis of pulmonary and EPTB. This tool has an important diagnostic value for detecting MTB in smear negative cases as it has out performed ZN microscopy by 10-15% in our study. It can increase the detection of MTB in EPTB by 2-3 times as compared to conventional techniques.

We suggest that in addition to its recommended use in MDR cases, its routine use may be extended to screening of smear negative patients with high suspicion of TB and for diagnosis of EPTB.

We further recommend, more such studies should be conducted to evaluate the feasibility of using this instrument in our local health care settings.

## Authors’ Contribution:

**SI: **Collection of cases, Data collection.

**AZ: **Running Gene-Expert, Practical work.

**SH:** Analysis of results of Gene-Expert.

**NWY: **Manuscript writing.

**MA:** Editing and revising the manuscript.

## References

[1] Helb D, Jones M, Story E, Boehme C, Wallace E, Ho K, Kop J (2010). Rapid detection of Mycobacterium tuberculosis and rifampin resistance by use of on-demand, near-patient technology. J Clin Microbiol.

[2] World Health Organization Executive Summary, Global Tuberculosis Report 2012.

[3] WHO (2008). Guidelines for the programmatic management of drug-resistant tuberculosis.

[4] (2009). Eastern Mediterranean Regional Office (World Health Organization). Cairo: STOP TB: TB situation in region - Country Profile Pakistan.

[5] Parveen K, Momen A, Kobra K (2011). Frequency of Tuberculosis in patients with enlarged lymph nodes and its cost-effective first line of diagnosis in our country. J Dhaka National Med Coll Hos.

[6] Saeed N (Nizamuddin S. Clinical manifestation of extrapulmonary tuberculosis. Med Chann. 2012). Islam A. Burki HB.

[7] Alvarez-Uria G, Azcona JM, Midde M, Naik PK, Reddy S, Reddy R (2012). Rapid diagnosis of pulmonary and extrapulmonary tuberculosis in HIV-infected patients Comparison of LED fluorescent microscopy and the geneXpert MTB/RIF assay in a district hospital in India. Tuber Res Treatment.

[8] Boehme CC, Nabeta P, Hillemann D, Nicol MP, Shenai S, Krapp F (2010). Rapid molecular detection of tuberculosis and rifampin resistance. N Eng J Med.

[9] Hillemann D, Rüsch-Gerdes S, Boehme C, Richter E (2011). Rapid molecular detection of extra pulmonary tuberculosis by the automated GeneXpert MTB/RIF system. J Clin Microbio.

[10] World Health Organization, Rapid Implementation of the Xpert MTB/RIF Diagnostic Test, World Health Organization (2011). http://whqlibdoc.who.int/publications/2011/9789241501569_eng.pdf.

[11] Armand S, Vanhuls P, Delcroix G, Courcol R, Lemaitre N (2011). Comparison of the Xpert MTB/RIF test with an IS6110-TaqMan real-time PCR assay for direct detection of Mycobacterium tuberculosis in respiratory and nonrespiratory specimens. J Clin Microbiol.

[12] Theron G, Peter J, van Zyl-Smit R, Mishra H, Streicher E, Murray S (2011). Evaluation of the Xpert MTB/RIF assay for the diagnosis of pulmonary tuberculosis in a high HIV prevalence setting. Am J Respir Crit Care Med.

[13] Zeka AN, Tasbakan S, Cavusoglu C (2011). Evaluation of the GeneXpert MTB/RIF assay for rapid diagnosis of tuberculosis and detection of rifampin resistance in pulmonary and extrapulmonary specimens. J Clin Microbiol.

[14] Vadwai V, Boehme C, Nabeta P, Shetty A, Alland D, Rodrigues C (2011). Xpert MTB/RIF: a new pillar in diagnosis of extrapulmonary tuberculosis?. J Clin Microbiol.

[15] International Union against Tuberculosis and Lung Disease (2000). Sputum examination for tuberculosis by direct microscopy in low income countries. Technical Guide.

[16] Chihota VN, Grant AD, Fielding K, Ndibongo B, van Zyl A, Muirhead D, Churchyard GJ (2010). Liquid vs solid culture for tuberculosis: performance and cost in a resource-constrained setting. Int J Tuber Lung Dis.

[17] Lawn SD, Nicol MP (2011). Xpert® MTB/RIF assay: development, evaluation and implementation of a new rapid molecular diagnostic for tuberculosis and rifampicin resistance. Fut Microbiol.

[18] Batz HG, Cooke GS, Reid SD (2011). Towards lab-free tuberculosis diagnosis. Treatment Action Group, the TB/HIV Working Group of the Stop TB Partnership, Imperial College, and the MSF Access Campaig.

[19] Chang K, Lu W, Wang J, Zhang K, Jia S, Li F (2012). Rapid and effective diagnosis of tuberculosis and rifampicin resistance with Xpert MTB/RIF assay: a meta-analysis. J Infect.

[20] Marlowe EM, Novak-Weekley SM, Cumpio J, Sharp SE, Momeny MA, Babst A (2011). Evaluation of the Cepheid Xpert MTB/RIF assay for direct detection of Mycobacterium tuberculosis complex in respiratory specimens. J Clin Microbiol.

[21] Moure R, Munoz L, Torres M, Santin M, Martin R, Alcaide F (2011). Rapid detection of Mycobacterium tuberculosis complex and rifampin resistance in smear-negative clinical samples by use of an integrated real-time PCR method. J Clin Microbiol.

[22] Bowles EC, Freyee B, van Ingen J, Mulder B, Boeree MJ, van SD (2011). Xpert MTB/RIF(R), a novel automated polymerase chain reaction-based tool for the diagnosis of tuberculosis. Int J Tuberc Lung Dis.

[23] Van Rie A, Menezes C, Scott L (2011). High yield, sensitivity and specificity of Xpert MTB/RIF for M. tuberculosis detection in fine needle aspirates from HIV-infected TB suspects; Program and abstracts of the 18th Conference on Retroviruses and Opportunistic Infections.

[24] Tortoli E, Russo C, Piersimoni C, Mazzola E, Dal Monte P, Pascarella M (2012). Clinical validation of Xpert MTB/RIF for the diagnosis of extrapulmonary tuberculosis. Eur Respir J.

[25] World Health Organization (2011). Policy Framework for Implementing TB Diagnostics.

[26] Vadwai V, Boehme C, Nabeta P, Shetty A, Alland D, Rodrigues C (2011). Xpert MTB/RIF: a new pillar in diagnosis of extrapulmonary tuberculosis?. J Clin Microbiol.

